# Species diversity revealed in *Sigmella* Hebard, 1929 (Blattodea, ectobiidae) based on morphology and four molecular species delimitation methods

**DOI:** 10.1371/journal.pone.0232821

**Published:** 2020-06-10

**Authors:** Meng Li, Qiongyao Zhao, Rong Chen, Jiajun He, Tao Peng, Wenbo Deng, Yanli Che, Zongqing Wang

**Affiliations:** Institute of Entomology, College of Plant Protection, Southwest University, Beibei, Chongqing, China; Nanjing Agricultural University, CHINA

## Abstract

Cockroaches are one of the major decomposers involved in biogeochemical cycles. Cockroaches have an amazing amount of diversity, but most of them remain unknown due to the shortage of the trained taxonomists and the limitations of morphology-based identification. We obtained 49 COI sequences (including 42 novel sequences) and 32 novel 28S sequences for 5 *Sigmella* morphospecies collected from 11 localities. Three are new to science: *Sigmella digitalis* sp. nov., *Sigmella exserta* sp. nov. and *Sigmella normalis* sp. nov. Based on four species delimitation methods (ABGD, GMYC, BINs and bPTP), a total of 6 molecular operational taxonomic units (MOTUs) were recovered for 5 morphospecies. These were then confirmed by tree building methods using COI and combined data (COI and 28S). We detected more than one MOTU in the morphospecies *S*. *digitalis* sp. nov., which can indicate genetic diversity. Detailed morphological evidence for each MOTU is provided to confirm these slight variations and we conclude that natural barriers are likely the main cause of genetic diversity.

## Introduction

Apart from a few species of Blattodea such as *Periplaneta americana*, *Periplaneta fuliginosa* and *Blattella germanica* that are domestic pests, most cockroaches play a major role as decomposers in biogeochemical cycles [1]. Generally speaking, the diversity of Blattodea is strongly underestimated owing to the lack of taxonomists [2]. And many species remain unknown or misidentified because of different juvenile morphology, sexual dimorphism and polymorphism [1, 3, 4] which cannot be easily resolved by only applying morphological characters. For some similar species, it is very challenging if only morphology-based identification is applied. For example, individuals of related *Sigmella* species have a highly conserved external morphology, but exhibit slight variations in the shape of the male genitalia, which comprises an impediment to judging the interspecific differences (Li M & Wang ZQ, personal observation).

Since Hebert *et al*. [5] came up with the concept, DNA barcodes have proven to be a reliable and cost-effective method to identify species in insect groups (Isoptera [6]; Coleoptera [7]; Orthoptera [8]; Odonata [9]) and to detect cryptic species [10, 11]. DNA barcoding has been applied to delimit Blattodea species [12–14] successfully. These studies have confirmed the importance of DNA barcodes when used in combination with other lines of evidence (morphology, chromosome numbers or locality) to performing molecular species delimitation. Four methods of molecular species delimitation (General Mixed Yule-coalescent (GMYC), Automatic Barcode Gap Discovery (ABGD), Poisson-Tree-Processes (bPTP) and Barcode Index Numbers (BINs)) have been widely used in discerning species as independently evolving lineages [15–19]. And in many groups of organisms, morphospecies often disguise considerable genetic diversity, indicating the existence of cryptic species [20].

Hebard [21, 22] established the genus *Sigmoidella* but then replaced *Sigmoidella* with *Sigmella* in 1940 since the name *Sigmoidella* was occupied. Bruijning [23] compared *Sigmella* and *Scalida* Hebard, 1929 using Hebard’s key, but failed to distinguish these two genera, and therefore synonymized *Sigmella* with *Scalida*. After examining the type species, *Scalida latiusvittata* and *Sigmella adversa*, Roth [24] concluded that both genera were valid on the basis of the characteristics: cubitus veins of *Sigmella* always more pronounced than *Scalida* and strong differences in subgenital and supra-anal plates. On that basis, Roth [24] transferred many species from *Scalida* to *Sigmella*. However, Wang & Che [25] moved two of them back to *Scalida* to support Bey-Bienko [26, 27] based on characteristics of the medial and cubital veins, the supra-anal plate, the style and the interstyle process. Up to now, 23 species of the genus *Sigmella* were known worldwide, of which, 4 species are from China.

Even with the help of DNA barcodes using the GMYC method, three *Sigmella* species were grouped together owing to the small genetic distance among them [13]. However, they should have been treated as different candidate species in light of the male genitalia, which highlights the need for the approaches to the identification of *Sigmella* species on a larger scale. Given this, we selected 49 individuals from 11 localities to represent the wide variations in male genitalia within morphospecies and to assess the delineation of *Sigmella* based on morphological evidence. We apply four species delimitation methods (GMYC, ABGD, BINs and bPTP) and choose the most congruent result to understand species limits and reveal the species diversity in *Sigmella* species. In order to confirm this delimitation result, an additional analysis using the combined dataset (COI and 28S) was performed to exhibit the reciprocal monophyletic criteria.

## Materials and methods

### Specimens

All cockroach specimens were collected at public area. We acquired specimens at eight different locations (outside the Nature Reserve) in Hainan Province and three locations (in the Nature Reserve) in Guangxi autonomous region, and confirm that all cockroach species are not endangered or protected species. *Sigmella* samples in this study were mostly collected on leaves but were also attracted by light at night. We collected at least 5 samples from different collecting locations. Specimens were stored in 100% ethanol and preserved at -20°C. All voucher specimens and type specimens were deposited in the Institute of Entomology, Southwest University (SWU). In total, we successfully obtained 42 *Sigmella* sequences from 11 sampling localities (Table 1) with the exception of a few samples or the failure in sequencing.

**Table 1:** Sample ID, voucher ID, species name, BOLD process IDs, GenBank accession numbers and collection data of *Sigmella* spp. used in this study. The letter f after the voucher indicates the sample is female.

No.	Voucher ID	Species	BOLD process ID	GenBank Accession Number		Locality	Collection Date
				COI	28S		
**1**	c1DLS1	*S*. *puchihlungi*	BOLD: ADL5122	MT394226	N.A.	Mt. Diaoluoshan, Lingshui, Hainan (N 18°43.498′ E 109°52.094′)	03 May, 2013
**2**	c1DLS2		BOLD: ADL5122	MT394227	N.A.		
**3**	c1DLS3		BOLD: ADL5122	MT394228	N.A.		
**4**	c1DLS4		BOLD: ADL5122	MT394229	N.A.		
**5**	cJFL1		BOLD: ADL5122	MT394230	N.A.	Mingfenggu, Mt. Jianfengling, Hainan	26–27 May, 2014
**6**	cJFL2		BOLD: ADL5122	MT394231	MT394270		
**7**	cJFL4		BOLD: ADL5122	MT394232	MT394271		
**8**	cJFL5		BOLD: ADL5122	MT394233	MT394272		
**9**	a1DLS1	*S*. *normalis* sp. nov.	BOLD: ADJ8144	MT394234	MT394273	Mt. Diaoluoshan, Lingshui, Hainan (N 18°43.430′ E 109°52.126′)	22–23 May, 2014
**10**	a1DLS2		BOLD: ADJ8144	MT394235	MT394274		
**11**	a1DLS3		BOLD: ADJ8144	MT394236	MT394275		
**12**	a1DLS4		BOLD: ADJ8144	MT394237	MT394276		
**13**	a1DLS5		BOLD: ADJ8144	MT394238	MT394277		
**14**	aLMS1		BOLD: ADJ8144	MT394239	MT394278	Mt. Limushan, Hainan (N 19°10.047′ E 109°44.988′)	16 Apr., 2015
**15**	aLMS2		BOLD: ADJ8144	MT394240	MT394279		
**16**	aLMS5		BOLD: ADJ8144	MT394241	MT394280		
**17**	aWZS1		BOLD: ADJ8144	MT394242	MT394281	Mt. Wuzhishan, Hainan (N 18°54.290′ E 109°41.087′)	18–21 May, 2014
**18**	aWZS2		BOLD: ADJ8144	MT394243	MT394282		
**19**	aWZS3		BOLD: ADJ8144	MT394244	MT394283		
**20**	aWZS4		BOLD: ADJ8144	MT394245	MT394284		
**21**	aWZS5		BOLD: ADJ8144	MT394246	MT394285		
**22**	aJFL1		BOLD: ADJ8144	MT394247	MT394286	Mingfenggu, Mt. Jianfengling, Hainan (N 19°05.176′ E 109°07.336′)	24 Apr., 2015
**23**	aJFL2		BOLD: ADJ8144	MT394248	MT394287		
**24**	aJFL3		BOLD: ADJ8144	MT394249	MT394288		
**25**	aJFL4		BOLD: ADJ8144	MT394250	MT394289		
**26**	aJFL5		BOLD: ADJ8144	MT394251	MT394290		
**27**	bBWL1	*S*. *digitalis* sp. nov.	BOLD: ADL5124	KY349526*	N.A.	Mt. Bawangling, Hainan (N 19°05.176′ E 109°07.336′)	29 Apr., 2015
**28**	bBWL2		BOLD: ADL5124	KY349527*	N.A.		
**29**	bBWL3		BOLD: ADL5124	KY349524*	N.A.		
**30**	bBWL4		BOLD: ADL5124	MT394252	N.A.		
**31**	bBWL5(f)		BOLD: ADL5124	MT394253	N.A.		
**32**	bLPC1		BOLD: ADL3982	KY349529*	MT394299	Liupancun, Jiyangzhen, Sanya, Hainan (N 18°14.846′ E 109°37.482′)	08 Apr., 2015
**33**	bLPC2		BOLD: ADL3982	KY349528*	MT394300		
**34**	bLPC3		BOLD: ADL3982	KY349530*	N.A.		
**35**	bLPC4		BOLD: ADL3982	MT394254	MT394301		
**36**	dSTS1	*S*. *exserta* sp. nov.	BOLD: ADL5125	KY349536*	N.A.	Mt. Shengtangshan, Jinxiu, Guangxi (N 23°58.414′ E 110°07.168′)	04–05 June, 2014
**37**	dSTS2		BOLD: ADL5125	MT394255	MT394291		
**38**	dSTS3		BOLD: ADL5125	MT394256	MT394292		
**39**	dSTS4		BOLD: ADL5125	MT394257	MT394293		
**40**	eSS1(f)	*S*. *schenklingi biguttata*	BOLD: ADL2943	MT394258	MT394294	Shiwandashan Park, Shangsi, Guangxi	28 June, 2015
**41**	eSS2		BOLD: ADL2943	MT394259	N.A.		
**42**	eSS3(f)		BOLD: ADL2943	MT394260	N.A.		
**43**	eSS4(f)		BOLD: ADL2943	MT394261	MT394295		
**44**	eSS5(f)		BOLD: ADL2943	MT394262	N.A.		
**45**	eGP1		BOLD: ADL2943	MT394263	MT394296	Longtan Park, Guiping, Guangxi	31 May-02 June, 2014
**46**	eGP2		BOLD: ADL2943	MT394264	N.A.		
**47**	eGP3(f)		BOLD: ADL2943	MT394265	N.A.		
**48**	eGP4		BOLD: ADL2943	MT394266	MT394297		
**49**	eGP5		BOLD: ADL2943	MT394267	MT394298		

* indicates data from Che et al. [13].

### Morphological types

We first examined all *Sigmella* samples mainly by morphological characters, including overall body shape and coloration, pronotal coloration and markings, the seventh abdominal tergum shape, the hind margin of supra-anal plate, as well as the subgenital plate. Male adults were then morphologically identified into morphospecies. Within each morphospecies, we chose male individuals sampled from different localities in order to obtain more genetic diversity. But for different variants from the same locality within the same types, we also attempted to sample for *Sigmella* diversity. Specimens of female adults were not identified due to the lack of diagnostic characters, but used directly for PCR analysis and DNA sequencing.

### DNA sequencing

Total DNA was extracted using TIANamp Genomic DNA Kit (Tiangen Biotech, Beijing), and stored at -20°C. Our PCR used universal or modified primers for COI (COI-F:5’-GGTCAACAAATCATAAAGATATTGG-3’**/**COI-R:5’-TAAACTTCAGGGTGACCAAAAAA TCA-3’; COI-F1: 5’-CTATCACCTATTACTCAGCCAT-3’**/**COI-R: 5’-TAAACTTCWGGRT GWCCAAARAATCA-3’) and universal primers for 28S (28S-F:5’-ACACGGACCAAGGAGTCTAAC-3’**/**28S-R:5’-GTCCTGCTGTCTTAAGCAACC-3’). Each PCR was performed in Analytik Jena EasyCycler with 25μL volumes including 14.25μL of ultrapure water, 2.5μL of 10 × buffer (Mg^2+^ Free), 2μL of MgCl_2_ (25 mM), 2μL of dNTP mixture, 1μL of each primer (F and R), 0.25μL of Taq polymerase, and 2μL of DNA template and followed cycling conditions: 5min at 95°C, followed by 35 cycles of 45s at 94°C, 45s at 45–55°C, 45s at 72°C (COI) and 1min at 95°C, 1min at 48–55°C, and 1min at 72°C (28S), followed by a final extension step at 72°C for 10min. The amplified samples were tested using agarose gel electrophoresis and sent for sequencing at BGI Technology Solutions Company Limited (BGI-Tech) (Beijing, China). Finally, we uploaded COI and 28S sequences at the National Center for Biotechnology Information (NCBI) GenBank (Table 1).

### Sequence processing and phylogenetic analyses

A total of 73 COI sequences were analyzed, including 42 *Sigmella* sequences from this study and 7 *Sigmella* sequences from Che et al. [13] (Table 1), 24 sequences representing 16 species of other cockroaches downloaded from GenBank, and 1 mantid species as the outgroup (KR148854) (Table 2). Sequences were aligned using MUSCLE 3.8 [28]. Among our 49 *Sigmella* sequences, 23 identical COI haplotypes were found and removed from this analysis. Intraspecific and interspecific genetic divergence values (COI) are quantified based on the Kimura 2-parameter (K2P) distance model [29], using MEGA 7 [30]. To test the successful identification rate of COI, we employed at least one sequence of 28S rRNA for samples from each locality with 38 total sequences (Table 1). The COI dataset was divided into 2 partitions by codon position (pos12, pos3), and PartitionFinder v1.1.1 [31] was used to determine the best fitting models for COI_pos12, COI_pos3 and 28S. Maximum Likelihood (ML) and Bayesian Inference (BI) analyses were used to explore the reciprocal monophyletic criteria for the species delimitation of these closely related species based on two datasets: the COI dataset and the combined dataset (COI and 28S). For ML, RAxML [32] was performed with the GTRGAMMA model for the datasets, and bootstrap values were implemented for 1000 replicates. For BI, MrBayes 3.2.6 [33] was used with the best fitting models as follows: COI_pos12, TrNef+I+G; COI_pos3, K81uf+G; 28S, TVMef+G. We ran two independent sets of Markov chains, each with one cold and three heated chains for 107 generations. Samples were drawn every 1000 steps and the first 25% were discarded as burn-in. When the average standard deviation of split frequencies was below 0.01, we inferred convergence.

**Table 2:** Blaberoidea and mantids (outgroup) in this study.

Species	Family	Accession Number	Reference
***Sorineuchora bivitta***	Ectobiidae	KY349592, KY349593	Che *et al*. [13]
***Sorineuchora nigra***	Ectobiidae	KY349519–KY349522	Che *et al*. [13]
***Allacta ornata***	Ectobiidae	KY349665	Che *et al*. [13]
***Balta jinlinorum***	Ectobiidae	KY349666–KY349669	Che *et al*. [13]
***Scalida ectobioides***	Ectobiidae	KM497412	Unpublished
***Scalia latiusvittata***	Ectobiidae	MT394268, MT394269	This study
***Minablatta* sp.**	Blaberidae	KP986424	Legendre *et al*. [34]
***Geoscapheus dilatatus***	Blaberidae	HQ936976	Unpublished
***Macropanesthia kinkuna***	Blaberidae	HQ936979	Unpublished
***Zetobora sp*.**	Blaberidae	KF372540	Legendre *et al*. [35]
***Gromphadorhina portentosa***	Blaberidae	KM577153	Von Beeren *et al*. [36]
***Rhabdoblatta bielawskii***	Blaberidae	KF640067	Yue *et al*. [37]
***Rhabdoblatta marginata***	Blaberidae	KF640068	Yue *et al*. [37]
***Epilampra* sp.**	Blaberidae	EU253831	Legendre et al.[38]
***Parasphaeria boleiriana***	Blaberidae	EU253832	Legendre *et al*. [38]
***Mantis religiosa***	Mantidae	KR148854	Hebert *et al*. [39]

We performed four molecular species delimitation methods based on COI data: Automatic Barcode Gap Discovery (ABGD) [17], the General Mixed Yule-coalescent (GMYC) [16], Poisson-Tree-Processes [18] (bPTP) and Barcode Index Numbers [19] (BINs), in order to estimate the number of molecular operational taxonomic units (MOTUs) from *Sigmella*.

The GMYC method requires a fully-resolved ultrametric tree for the analysis to define species. Time-resolved gene trees were inferred with BEAST 1.8.1 [40] using the best models from PartitionFinder V1.1.1 under the following settings: rate variation was modeled among branches using a strict clock model with the mean clock rate fixed to 1, and the Birth-Death speciation was used as a tree prior. We then applied the GMYC method to the ultrametric gene tree using the SPLITS package [41] in R [42]. The species delimited were compared to a one species null model using a likelihood ratio test. Automatic Barcode Gap Discovery (ABGD) is available at web interface (http://wwwabi.snv.jussieu.fr/public/abgd/) and was used as a simple, quick and efficient method with the default settings by Jukes-Cantor (JC69) and p distance model with relative gap width (X = 1.0). BINs were assigned automatically on BOLD workbenchv4.0 (http://www.boldsys-tems.org; analyzes performed on 20 March 2018). For bPTP, a Maximum Likelihood (ML) tree was generated from COI data in RAxML and then used with the default setting at the species delimitation web server (http:// species.h-its.org/ptp/).

### Nomenclatural acts

The electronic edition of this article conforms to the requirements of the amended International Code of Zoological Nomenclature, and hence the new names contained herein are available under that Code from the electronic edition of this article. This published work and the nomenclatural acts it contains have been registered in ZooBank, the online registration system for the ICZN. The ZooBank LSIDs (Life Science Identifiers) can be resolved and the associated information viewed through any standard web browser by appending the LSID to the prefix "http://zoobank.org/". The LSID for this publication is: urn:lsid:zoobank.org:pub:61470EE8-7A51-480C-8F5A-877FB149B039. The electronic edition of this work was published in a journal with an ISSN, and has been archived and is available from the following digital repositories: PubMed Central, LOCKSS [Researchgate].

## Results

### Morphological delimitation of *Sigmella*

**Fig 1:**
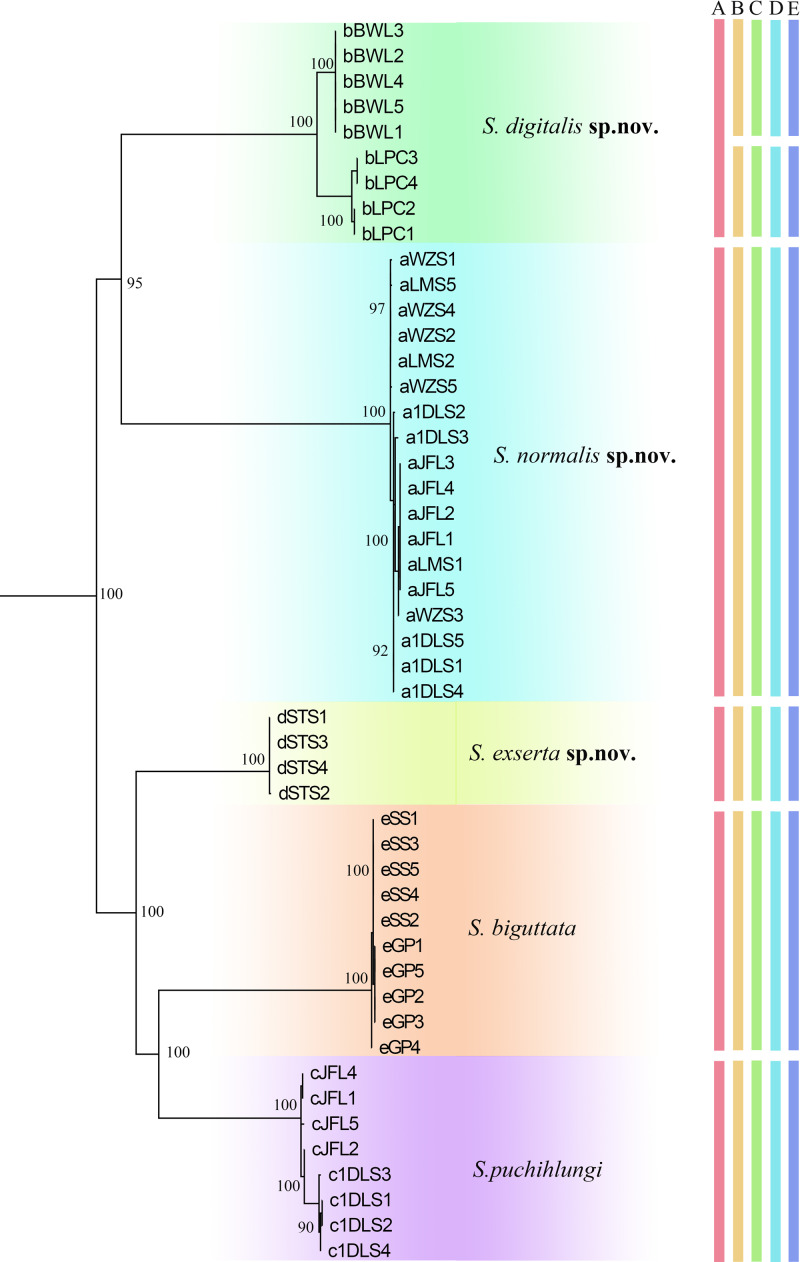
Maximum Likelihood (ML) gene tree with delimited MOTUs of the studied 5 *Sigmella* morphospecies. Numbers near node indicate the maximum-likelihood bootstrap values. The five morphospecies: *S*. *schenklingi biguttata*, *S*. *digitalis* sp. nov., *S*. *normalis* sp. nov., *S*. *puchihlungi* and *S*. *exserta* sp. nov. were highlighted by color corresponding to the clade. Letter indicates delimited MOTUs by different methods (A: Morphospecies, B: ABGD, C: GMYC, D: BINs, E: bPTP).

On the basis of morphological characters including male genitalia, we were able to identify 5 morphospecies of *Sigmella* among the 49 samples that we examined (Fig 1). Herein three new species, *S*. *digitalis* sp. nov., *S*. *exserta* sp. nov. and *S*. *normalis* sp. nov. are established, and two known species, *S*. *puchihlungi* and *S*. *schenklingi biguttata* are well identified according to only morphological characters including male genitalia (body color, the maculae on pronotum, the saclike glands of the seventh abdominal tergum, the characteristics of supra-anal and subgenital plate) (Figs 3A–3H and S4A–S4R). Species descriptions are provided below. All the samples of *Sigmella digitalis* sp. nov. (with light green highlights in Fig 1) show a range of slight variations in male genitalia: the fingerlike glands of the seventh abdominal tergum with apex more or less tapering (Fig 3E3) or blunt (Fig 3F3), the seventh abdominal tergum with hind margin concave at middle (Fig 3E3) or straight (Fig 3F3), the spines situated near the hind margin of the supra-anal plate slender and unbifurcated (Fig 3E2) or robust and bifurcated (Fig 3F2), the straight process arising on subgenital plate with two spines extending beyond the end (Fig 3E1) or not (Fig 3F1), and the posterolateral border of subgenital plate with 3 large spines (Fig 3E1) or 4 small spines (Fig 3F1). It is rather challenging and confusing to distinguish them based only on morphological characters even with the male genitalia information, so we temporarily treat them as intraspecific variations.

### Phylogenetic analysis based on COI and the combined dataset

In this study, we acquired 42 COI sequences, whose length excluding primers was 658 bp, plus 32 28S sequences with the length of 713 bp. All new sequences have been deposited in GenBank with accession numbers MT394226 to MT394268 for COI, and MT394269 to MT394298 for 28S (Tables 1 and 2). The COI sequences that we sequenced have rich AT content (62.6%). Sequence analysis revealed that 290 sites were variable, of which 264 were parsimony informative. The 28S sequences have a high CG content (53.5%), and 349 sites were variable, of which 192 were parsimony informative.

Two phylogenetic methods (ML and BI) based on COI data revealed similar tree topologies but differed at deep phylogenetic levels, and the bootstrap values in ML (mostly MLB = 100) (Fig 1) were much higher than those in the BI tree (S1 Fig). In both the ML and BI analyses, the clades from reciprocal morphological groups including females constituted monophyletic groups with high support values. All *Sigmella* species were recovered as a monophyletic group, although tree topologies were not totally consistent across the different phylogenetic methods. The concatenated COI and 28S sequences were also used to test the utility of COI analysis (S2–S3 Figs), and both ML and BI analyses revealed similar topologies for most clades, although it was not totally consistent with that of COI data.

5 *Sigmella* morphospecies formed monophyletic groups as recovered in BI and ML analyses for COI and combined datasets (Fig 1 and S1–S3 Figs) with high support values (nearly 100).

### MOTUs estimation using different species delimitation methods

We used four molecular species delimitation methods (BINs, ABGD, GMYC, and bPTP) in our study to delimit the confusing *Sigmella* samples.

ABGD analysis for MOTUs detection was estimated with JC69 and P = 0.004642, 0.007743, 0.012915 and 0.021544 respectively and performed 6 MOTUs. BIN analysis of 49 sequences recovered 6 MOTUs (Table 1). The likelihoods of the null and GMYC models from COI analysis were 87.65596 and 131.6793 respectively. The GMYC was an improvement over the null model, and was clustered into 7 (confidence interval: 7–9) entities (likelihood ratio = 88.04658) including 6 *Sigmella* MOTUs, 16 other cockroach species and 1 mantid (outgroup taxa). The bPTP analysis has estimated 6 MOTUs in our COI dataset. This method produced additional MOTUs in one morphospecies, *S*. *digitalis* sp. nov. (2 MOTUs). These four methods have yielded almost identical results using COI data: only one MOTU for four morphospecies, *S*. *puchihlungi*, *S*. *schenklingi biguttata*, *S*. *exserta* sp. nov. and *S*. *normalis* sp. nov was detected, and two MOTUs were detected in *S*. *digitalis* sp. nov. Finally, a total of 6 MOTUs was recovered after assessing the results of four molecular species delimitation methods combined with morphological data. The intraMOTU and interMOTU sequence divergence of 6 *Sigmella* MOTUs ranged from 0.0 to 1.20% and 4.2 to 16.59%, respectively (Table 3 and S3 Table). For the additional MOTUs, the intraspecific K2P distances were considerably higher than the average intraspecific distances of the dataset indicating that these additional MOTUs exhibit considerable genetic diversity, and are more likely to represent cryptic species.

**Table 3:** K2P genetic distance among 6 *Sigmella* MOTUs.

MOTUs	K2P genetic distance				
***S*. *puchilungi***					
***S*. *normalis* sp. nov.**	0.1438				
***S*. *digitalis* sp. nov. (BWL)**	0.1428	0.1490			
***S*. *digitalis* sp. nov. (LPC)**	0.1498	0.1511	0.0418		
***S*. *exserta* sp. nov.**	0.1069	0.1352	0.1341	0.1473	
***S*. *schenklingi biguttata***	0.1275	0.1659	0.1460	0.1448	0.1360

**Fig 2:**
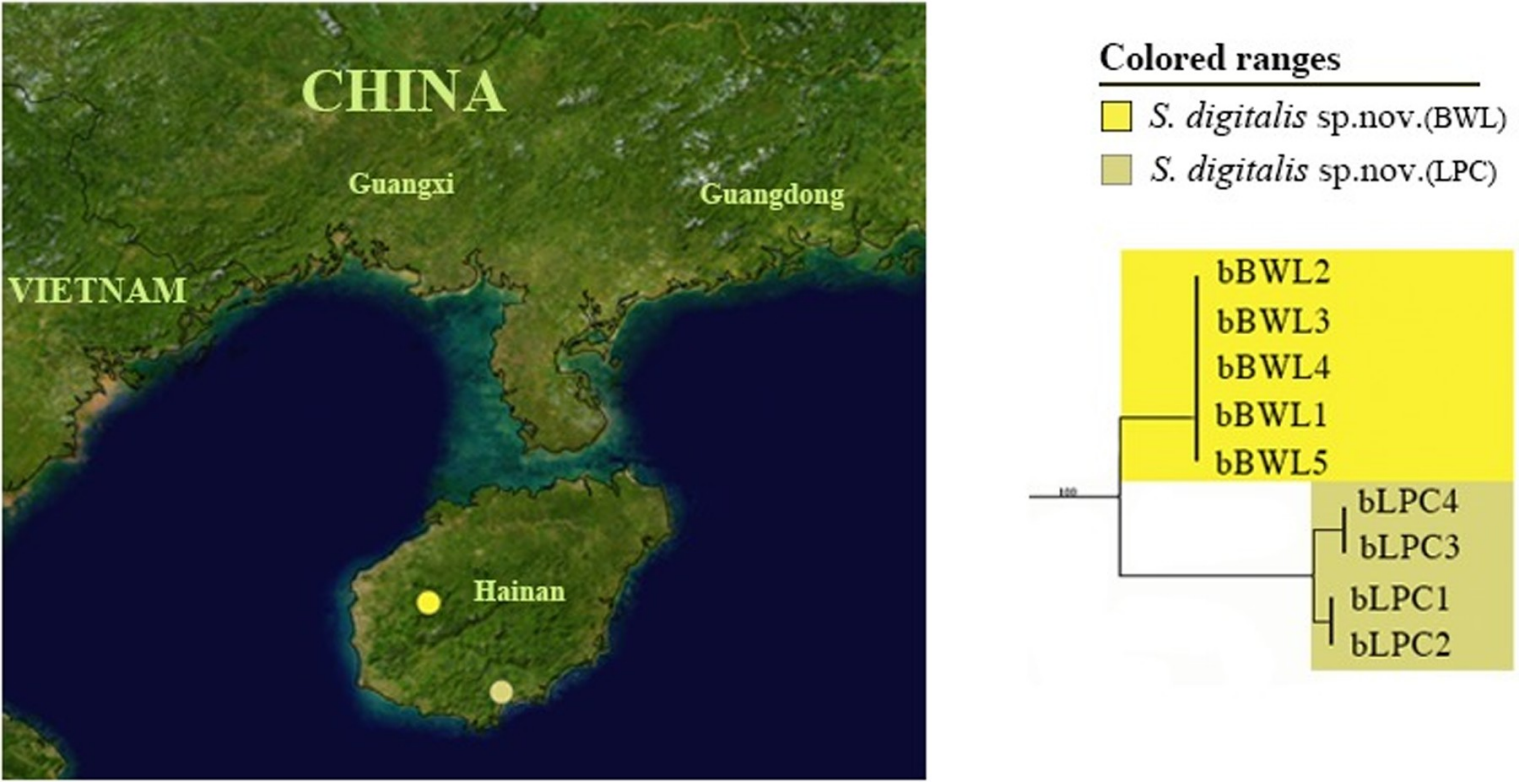
Pruned ML tree showing cryptic diversity of *S*. *digitalis* sp. nov. with MOTUs nomenclature and geographical locations. The map originates from “Blue Marble: Land Surface, Shallow Water, and Shaded Topography” (Visible Earth, NASA); all modifications were performed using Adobe Photoshop CS6.

**Fig 3:**
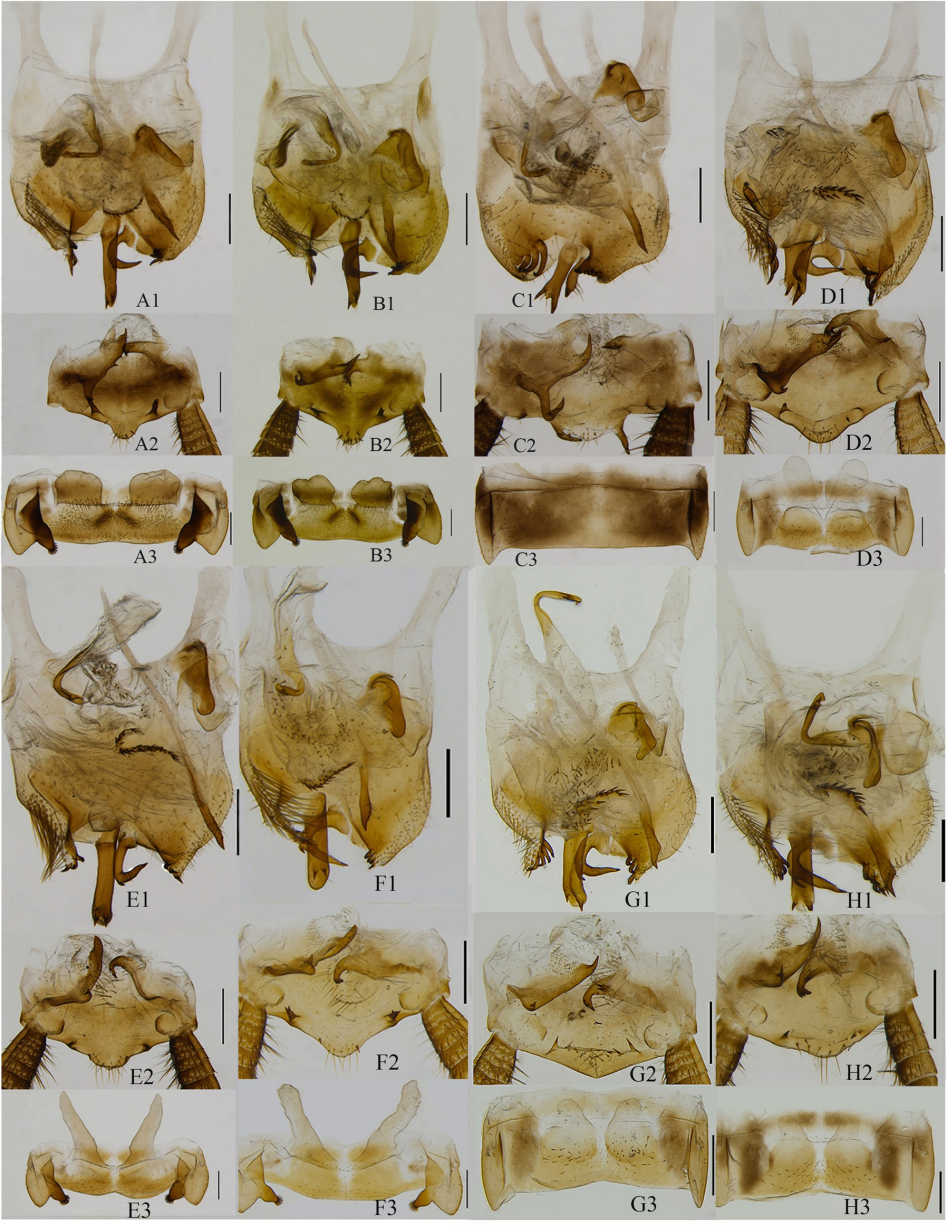
Male genitalia. *Sigmella puchihlungi*: A1–A3 (voucher c1DLS2), B1–B3 (voucher cJFL4). *S*. *normalis* sp. nov.: C1–C3 (voucher aWZS2). *S*. *exserta* sp. nov.: D1–D3 (voucher dSTS4). *S*. *digitalis* sp. nov.: E1–E3 (voucher bBWL1). *S*. *digitalis* sp. nov.: F1–F3 (voucher bLPC1). *Sigmella schenklingi biguttata*: G1–G3 (voucher eGP4), H1–H3 (voucher eSS2). A1–H1: subgenital plate, dorsal view; A2–H2: supra-anal plate, ventral view; A3–H3: the seventh abdominal tergum, ventral view. Scale = 0.5cm.

For the morphospecies, *S*. *digitalis* sp. nov., analysis based on both COI and combined datasets (COI and 28S) revealed two MOTUs, which formed two distinct clades in ML and BI trees (Figs 1 and S1–S3). Two clades of *S*. *digitalis* sp. nov. corresponded to two MOTUs (the K2P genetic distance: 0.0422), which were recovered in all four delimitation methods (Fig 1). These two MOTUs represent two different geographical locations from Hainan Province with 5 specimens (BWL) and 4 specimens (LPC), respectively (Fig 2). The intraclade K2P distances of *S*. *digitalis* sp. nov. (BWL) was 0.0 and for the other, 0.0051 (S3 Table); and K2P genetic distance between them was 0.0418 (Table 3). Morphologically, two clusters (BWL and LPC) of *S*. *digitalis* sp. nov. show no variation in body color, size and shape. But we could find some delicate morphological differences between the specimens of these two clusters: 1) the fingerlike glands of the seventh abdominal tergum with apex more or less tapering in the former (Fig 3E3), but the latter, blunt (Fig 3F3); 2) the seventh abdominal tergum with hind margin concave at middle (Fig 3E3), however, straight in the latter (Fig 3F3); 3) the spines situated near the hind margin of supra-anal plate slender and unbifurcated (Fig 3E2), but the latter with the spines robust and bifurcated (Fig 3F2); 4) the straight process arising on subgenital plate with two spines extending beyond the end (Fig 3E1), the other, not beyond the end (Fig 3F1); 5) the posterolateral border of subgenital plate with 3 large spines (Fig 3E1), but the latter with 4 small spines (Fig 3F1). Slight morphological differences exist between the two clusters; however, they were not readily distinguished and only determined as variations in morphology. Although two MOTUs were detected in *S*. *digitalis* sp. nov. by four molecular species delimitation methods, we did not recover the morphospecies, *S*. *digitalis* sp. nov., as a candidate for cryptic diversity when combined with morphological data.

### Establishment of three new species

On the basis of morphological characters, we were able to identify five *Sigmella* morphospecies including three new species among the 49 samples from 11 localities that we examined: *S*. *normalis* sp. nov., *S*. *digitalis* sp. nov. and *S*. *exserta* sp. nov.

### Key to species of *Sigmella* from China (based on males examined)

1. The seventh abdominal tergum unspecialized…*Sigmella normalis* sp. nov.
  - The seventh abdominal tergum specialized with a pair of glands….2
2. The seventh abdominal tergum with thick protuberance on posterolateral corners…3
  - The seventh abdominal tergum without thick protuberance on posterolateral corners…4
3. The seventh abdominal tergum with a pair of stubby glands… *S*. *puchihlungi*
  - The seventh abdominal tergum with a pair of long fingerlike glands… *Sigmella digitalis* sp. nov.
4. Glands long, beyond the anterior margin of seventh tergum… *Sigmella exserta* sp. nov.
  - Glands short, not beyond the anterior margin of seventh tergum… *Sigmella schenklingi biguttata*

### *Sigmella normalis* Li et Wang sp. nov.

urn:lsid:zoobank.org:act:C4BF6822-DD96-41DF-A17B-F9C286D8450A

(Figs 3C and S4E–S4F)

*Type materials*. **Holotype (SWU).** Male, Mt. Wuzhishan, Hainan, China, 18°54.290′ N, 109°41.087′ E, 795m, 18–21 May 2014, Shun-Hua Gui, Xin-Ran Li, Jian-Yue Qiu leg. **Paratypes.** 4 male, same data as holotype; 5 male, Mt. Diaoluoshan, Lingshui, Hainan, China, 18°43.430′ N 109°52.126 ′ E, 22–23 May 2014, Shun-Hua Gui, Xin-Ran Li, Jian-Yue Qiu leg. 3 male, Mt. Limushan, Hainan, China, 19°10.047′N, 109°44.988′E, Xin-Ran Li, Zhi-Wei Qiu leg. 5 male, Mingfenggu, Mt. Jianfengling, Hainan, China, 19°05.176′N, 109°07.336′ E, 24 April 2015, Lu Qiu, Qi-Kun Bai leg.

*Description*. Measurements (mm). Overall length including tegmen: male 12.4–14.2; pronotum length×width, male 2.4–2.8×3.0–3.7; tegmen length, male 10.7–11.8.

*Diagnosis*. The seventh abdominal tergum unspecialized (Fig 3C3); the hind margin of subgenital plate with two spine-like processes (Fig 3C1); the left style absent and the right style straight. Using these traits, *S*. *normalis* sp. nov. can be distinguished from its congeneric species.

*Male*. Body blackish brown. Vertex and face blackish brown. Base of antennae yellowish brown, the rest blackish brown. The fourth and fifth segment maxillary palpomere blackish brown, the rest yellow. Pronotal disk blackish brown or brown, the middle with an inconspicuous longitudinal, yellowish brown region. Tegmina yellowish brown, hind-wing longitudinal veins brown blackish brown. Abdominal terga brownish yellow (S4E–S4F Fig).

Interocular space narrower than the distance between antennal sockets. The fourth and fifth segment of maxillary palpus same in length, slightly shorter than the third. Pronotum subelliptical, posterior margin slightly convex medially. Tegmina and wings fully developed, extending beyond end of abdomen. M of tegmina with two branches. Hind-wing RA and RP parallel and inflated, M without branches, CuA with two complete branches, apical triangle evident. Front femur Type B_3_, pulvilli on four proximal tarsomeres, tarsal claws symmetrical, unspecialized, arolia present. The seventh abdominal tergum unspecialized (Fig 3C3).

Supra-anal plate symmetrical, hind margin weakly convex medially, two lateral margins with two spine-like processes; the left paraproct with two branches, the apex acute, and the left branch bent upward; the right paraproct with two to three small spines at the apex (Fig 3C2). Subgenital plate with asymmetrical, hind margin deeply excavated, two lateral margins with some brown spines, a large, straight process arising on dorsal surface and its apex with two spine-like small branches (Fig 3C1). The left style absent. The right style curved to right, the left side with a spine. Hooked phallomere (L3) on the right side. L2vm rod-like, apex acute. R3 consisting of two sclerites (Fig 3C1).

*Female*. Unknown.

*Distribution*. China (Hainan).

*Etymology*. The specific name is derived from Latin “*normalis*”, referring to the seventh abdominal tergum being unspecialized (Fig 3C3).

*Remarks*. This species resembles *S*. *puchihlungi* [43] in morphology, but differs from the latter in the following characteristics: (1) the former with unspecialized seventh abdominal tergum (Fig 3C3), while in the latter, specialized seventh abdominal tergum has swollen posterolateral corners and a thick protuberance on their inner margins, the middle with a pair of saclike glands, not exceeding the anterior margin (Fig 3A3 and 3B3); (2) the process of the subgenital plate with two big brown spines at apex (Fig 3C1), but in the latter, with two small spines at apex (Fig 3A1 and 3B1); (3) the former with right style straight (Fig 3C1); while the latter bent (Fig 3A1 and 3B1). Although this species is also similar to *S*. *balikpapanensis* in appearance, the first abdominal tergum being specialized or not can be helpful in distinguishing them (*S*. *balikpapanensis*: the first abdominal tergum specialized with a small posteromedial arch).

### *Sigmella digitalis* Li et Wang sp. nov.

urn:lsid:zoobank.org:act:8066A744-85BF-42F0-985A-8742AEB5C4D1

(Figs 3E–3F and S4G–S4L)

*Type materials*. **Holotype (SWU).** Male, Mt. Bawangling, Hainan, China, 19°05.176′ N, 109°07.336′ E, 29 April 2015, Lu Qiu, Qi-Kun Bai leg. **Paratypes**. 4 male,1female, same data as holotype; 4 male, Liupancun, Jiyangzhen, Sanya, Hainan, China, 18°14.846′N 109°37.482′E, 8 April 2015, Xin-Ran Li, Zhi-Wei Qiu leg.

*Description*. Measurements (mm). Overall length including tegmen: male 13.0–14.1; pronotum length×width, male 2.6–3.4×2.9–4.1; tegmen length, male 10.7–11.6.

*Diagnosis*. The seventh abdominal tergum with swollen posterolateral corners and thick protuberance on their inner margins, the middle with a pair of slim, long and fingerlike saclike glands, exceeding the anterior margin of abdominal tergum (Fig 3E3 and 3F3); the left style absent. On basis of these traits listed above, *S*. *digitalis* sp. nov. can be easily identified.

*Male*. Body yellowish brown. Vertex and face yellowish brown. Base of antennae yellowish brown, the rest blackish brown. Pronotal disk yellowish brown, without stripes or the posterior margin with two black dots. Tegmina yellowish brown (S4G and S4L Fig).

Interocular space narrower than the distance between antennal sockets. The fourth and fifth segment of maxillary palpus same length, slightly shorter than the third. Pronotum subelliptical, anterior margin truncate, posterior margin slightly convex medially. Tegmina and wings fully developed, extending beyond end of abdomen. Hind-wing RA and RP parallel and inflated, M bent medially without branches, CuA bent medially with three to four complete branches and two to four incomplete branches, apical triangle evident. Front femur Type B_3_, pulvilli on four proximal tarsomeres, tarsal claws symmetrical, unspecialized, arolia present. The seventh abdominal tergum specialized with swollen posterolateral corners and a thick protuberance on their inner margins, the middle with a pair of slim, long and fingerlike saclike glands, exceeding the anterior margin of seventh abdominal tergum (Fig 3E3 and3F3).

Supra-anal plate symmetrical, hind margin obviously convex medially, two lateral margins with two small spine-like processes; the left paraproct with two branches, the apex acute, the left branch small; the right paraproct with several spines at the apex (Fig 3E2 and 3F2). Subgenital plate with asymmetrical, hind margin deeply excavated, two lateral margins with some brown spines, a large, straight process arising on dorsal surface and its apex with two spine-like small branches. The left style absent. The right style curved to right, the left side with a spine. Hooked phallomere (L3) on the left side. L2vm rod-like, apex acute. R3 consisting of two sclerites (Fig 3E1 and 3F1).

*Female*. Similar to males in appearance. Hind margin of subgenital plate round, without concavity.

*Distribution*. China (Hainan).

*Etymology*. Latin term “*digitalis*” means fingerlike and refers to a pair of slim, long and fingerlike saclike glands present in the seventh abdominal tergum (Fig 3E3 and 3F3).

*Remarks*. This species resembles *S*. *puchihlungi* and *S*. *sipitanga*, but can be distinguished by the following characteristics: the seventh abdominal tergum with a pair of slim, long and fingerlike saclike glands, exceeding the anterior margin (Fig 3E3 and 3F3); but for the latter two species, the seventh abdominal tergum of *S*. *puchihlungi* with a pair of half-kidney-shaped saclike glands, not exceeding the anterior margin (Fig 3A3 and 3B3), *S*. *sipitanga* with a pair of distinct fossae separated by longitudinal ridge.

### *Sigmella exserta* Li et Wang sp. nov.

urn:lsid:zoobank.org:act:C72DBFB6-9F07-4354-992B-5FF5A7F85475

(Figs 3D and S4M–S4N)

*Type materials*. **Holotype (SWU).** Male, Mt. Shengtangshan, Jinxiu, Guangxi, China, 23°58.414′ N, 110°07.168′ E, 1182m, 04–05 June 2014, Shun-Hua Gui, Xin-Ran Li leg. **Paratypes.** 4 Male, same data as holotype.

*Description*. Measurements (mm). Overall length including tegmen: male 15.7–16.0; pronotum length×width, male 2.7–3.5×3.8–4.1; tegmen length, male 13.3–13.8.

*Diagnosis*. The strong and long saclike glands of seventh abdominal tergum exceeding the anterior margin of seventh abdominal tergum (Fig 3D3); the left style absent. Using these characteristics, *S*. *exserta* sp. nov. can be distinguished from other species within this genus.

*Male*. Body yellowish brown. Face yellow or yellowish brown. Base of antennae yellowish brown, the rest blackish brown. The fifth segment maxillary palpomere blackish brown, the rest yellowish brown. Pronotal disk yellowish brown, without stripes or the posterior margin with two black dots. Tegmina yellowish brown, hind-wing blackish brown. Abdominal sterna yellow or yellowish brown, the lateral margins with small blackish brown spots. Abdominal terga blackish brown (S4M and S4N Fig).

Interocular space narrower than the distance between antennal sockets. The fourth and fifth segment of maxillary palpus same length, slightly shorter than the third. Pronotum subelliptical, anterior margin truncate, posterior margin slightly convex medially. Tegmina and wings fully developed, extending beyond end of abdomen. Hind-wing RA and RP parallel and inflated, M bent medially without branches, CuA bent medially with two to four complete branches and one to two incomplete branches, apical triangle evident. Front femur Type B_3_, pulvilli on four proximal tarsomeres, tarsal claws symmetrical, unspecialized, arolia present. The seventh abdominal tergum specialized, the middle with a pair of strong and long saclike glands, exceeding the anterior margin of seventh abdominal tergum (Fig 3D3).

Supra-anal plate symmetrical, hind margin convex medially; the left paraproct with two branches, the apex acute, the left branch small; the right paraproct with two to three small spines at the apex (Fig 3D2). Subgenital plate asymmetrical, hind margin deeply excavated, two lateral margins with some brown spines, a large, straight process arising on dorsal surface and its apex with two spine-like branches. The left style absent. The right style curved to right, apex with a spine on the left side. Hooked phallomere (L3) on the left side. L2vm rod-like, apex acute. R3 consisting of two sclerites (Fig 3D1).

*Female*. Unknown.

*Distribution*. China (Guangxi).

*Etymology*. The Latin “*exsertus*” means projecting or long, referring to the saclike glands not exceeding the anterior margin of seventh abdominal tergum (Fig 3D3).

*Remarks*. This species resembles *S*. *schenklingi biguttata* in appearance, but it can be distinguished by the saclike glands of seventh abdominal tergum.

### *Sigmella schenklingi biguttata* (Bey-Bienko, 1954)

*Scalida biguttata* Bey-Bienko, 1954: 19.

*Sigmella schenklingi biguttata*, Princis 1969: 802.

Remark. Bey-Bienko(1954) [44] erected *Scalida biguttata* with two subspecies, *Scalida biguttata biguttata* and *Scalida biguttata unicolor*. Princis (1969) synomymized *Scalida biguttata unicolor* Bey-Bienko,1954 with *Sigmella schenklingi* (Karny,1915), and regarded *biguttata* as subspecies of *S*. *schenklingi*. Roth (1991) misunderstood Princis (1969: 802) [45] and regarded *Scalida biguttata biguttata* as the synonym of *Sigmella schenklingi*. So it is more reasonable to maintain the present taxonomic status before the type materials of these two subspecies are checked.

## Discussion

In this study, we examined the utility of using DNA barcode data in species identification and the assessing the genetic diversity in 5 morphospecies of *Sigmella* cockroaches recovered from our GMYC, BINs, bPTP and ABGD analysis. Using these methods for COI data, our study revealed the genetic uniqueness of 1 morphospecies (*S*. *digitalis* sp. nov.: 2 MOTUs). Our results therefore show that DNA-based species delimitation methods perform well for these morphologically similar and related cockroaches.

**Genetic diversity.** Our barcoding study revealed the genetic diversity in one *Sigmella* species, *S*. *digitalis* sp. nov. MOTUs were recovered by tree building methods and four automatic delimitation methods, but not ascertained by similar morphological characters, which might be due to incomplete lineage sorting of ancestral mitochondrial DNA polymorphisms, or an introgression of mitochondrial DNA causing genetic variability as occurs in Denticollinae beetles [46]. *S*. *digitalis* sp. nov. were collected from two different localities (BWL and LPC) in Hainan Province which are about 150 km distant but isolated by mountains. Geographic separation prevented gene flow between them and as a result, the high genetic distances existing between them (0.042) indicates the possibility of cryptic species. Morphological identification shows that slight morphological differences exist between the two clusters; however, they are not well distinguished and are only considered to be variations in morphology. Therefore, *S*. *digitalis* sp. nov. should not be recovered as a candidate for cryptic species.

## Conclusion

Our study shows that the molecular species delimitation methodology generates species hypotheses for cockroaches that are nearly consistent with those based on morphological techniques. Although it is tenuous to only apply these methods to delimit *Sigmella* species, molecular species delimitation analysis can play an important role in the discovery of genetic diversity and promises to be a rapid, precise, independent identification approach for pairing males with females to some extent. Moreover, as our study revealed, we can combine molecular species delimitation methods with morphological data to detect more MOTUs in *S*. *digitalis* sp. nov.; these approaches help us to understand cockroach biodiversity. Considering the lack of taxonomists with cockroach expertise, this phylogenetic inference of COI combined with molecular species delimitation methods proves to be an effective tool for the species delineation of *Sigmella* and the discovery of genetic diversity.

## Supporting information

S1 Fig Bayesian Inference (BI) tree derived from the COI gene. Outgroups are not shown. Numbers near node indicate the Bayesian posterior probabilities.
(TIF)

S2 Fig Maximum Likelihood (ML) tree derived from COI and 28S rRNA genes. Outgroups are not shown. Numbers near node indicate the maximum-likelihood bootstrap values.
(TIF)

S3 Fig Bayesian Inference (BI) tree derived from COI and 28S rRNA genes. Outgroups are not shown. Numbers near node indicate the Bayesian posterior probabilities.
(TIF)

S4 Fig Habitus. *Sigmella puchihlungi*: A–B (male, voucher cJFL5), C–D (female, voucher c3DLS2). *S*. *normalis* sp. nov.: E–F (male, voucher aWZS3). *S*. *digitalis* sp. nov.: G–H (male, voucher bBWL3), I–J (female, voucher bBWL2), K–L (male, voucher bLPC4). *S*. *exserta* sp. nov.: M–N (male, voucher dSTS4). *S*. *schenklingi biguttata*: O–P (female, voucher eSS1), Q–R (male, voucher eGP5). (A, C, E, G, I, K, M, O, Q) dorsal view; (B, D, F, H, J, L, N, P, R) ventral view; scale = 1cm.
(TIF)

S1 Table K2P genetic distances among 5 *Sigmella* morphospecies. (DOCX)

S2 Table K2P genetic distances within 5 *Sigmella* morphospecies. (DOCX)

S3 Table K2P genetic distances within 6 *Sigmella* MOTUs. (DOCX)
