# Supplementary figures and images for: Species diversity revealed in Sigmella Hebard, 1929 (Blattodea, ectobiidae) based on morphology and four molecular species delimitation methods
Source: PLoS One. 2020 Jun 10;15(6):e0232821. doi: 10.1371/journal.pone.0232821 (PMC7286484; doi:10.1371/journal.pone.0232821)

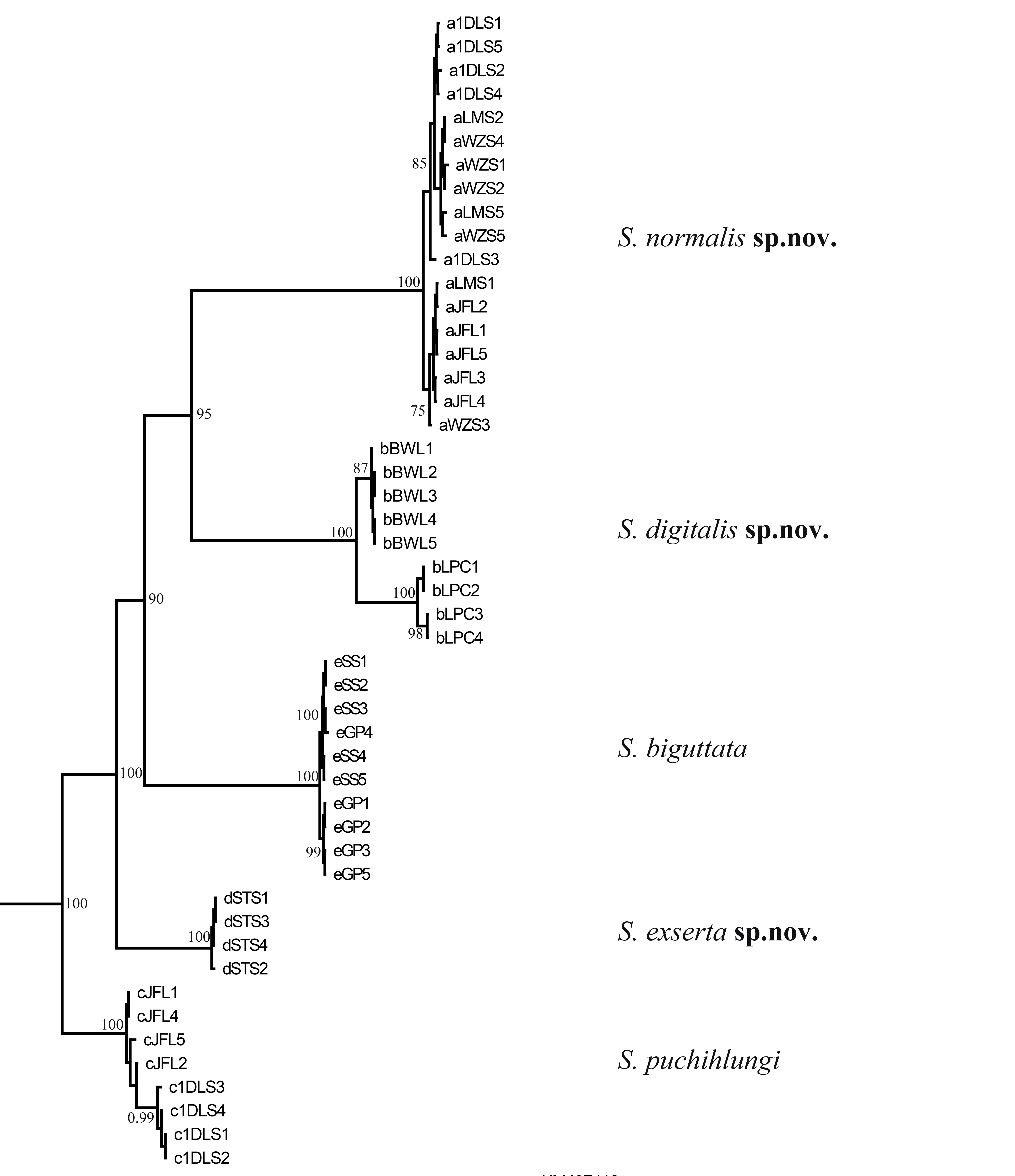

Supplement: S1 Fig — Outgroups are not shown. Numbers near node indicate the Bayesian posterior probabilities. (TIF) [file pone.0232821.s001.tif]

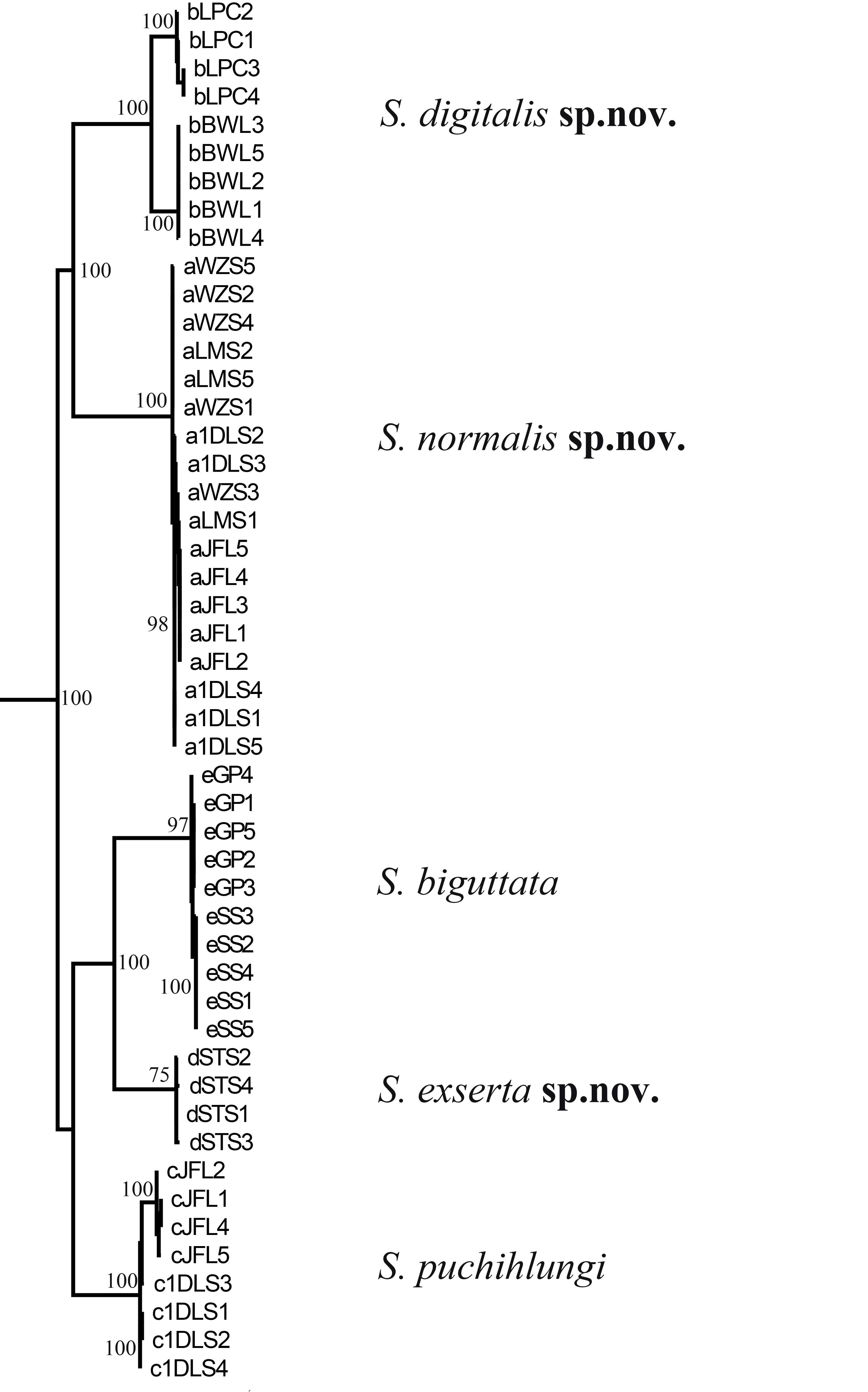

Supplement: S2 Fig — Outgroups are not shown. Numbers near node indicate the maximum-likelihood bootstrap values. (TIF) [file pone.0232821.s002.tif]

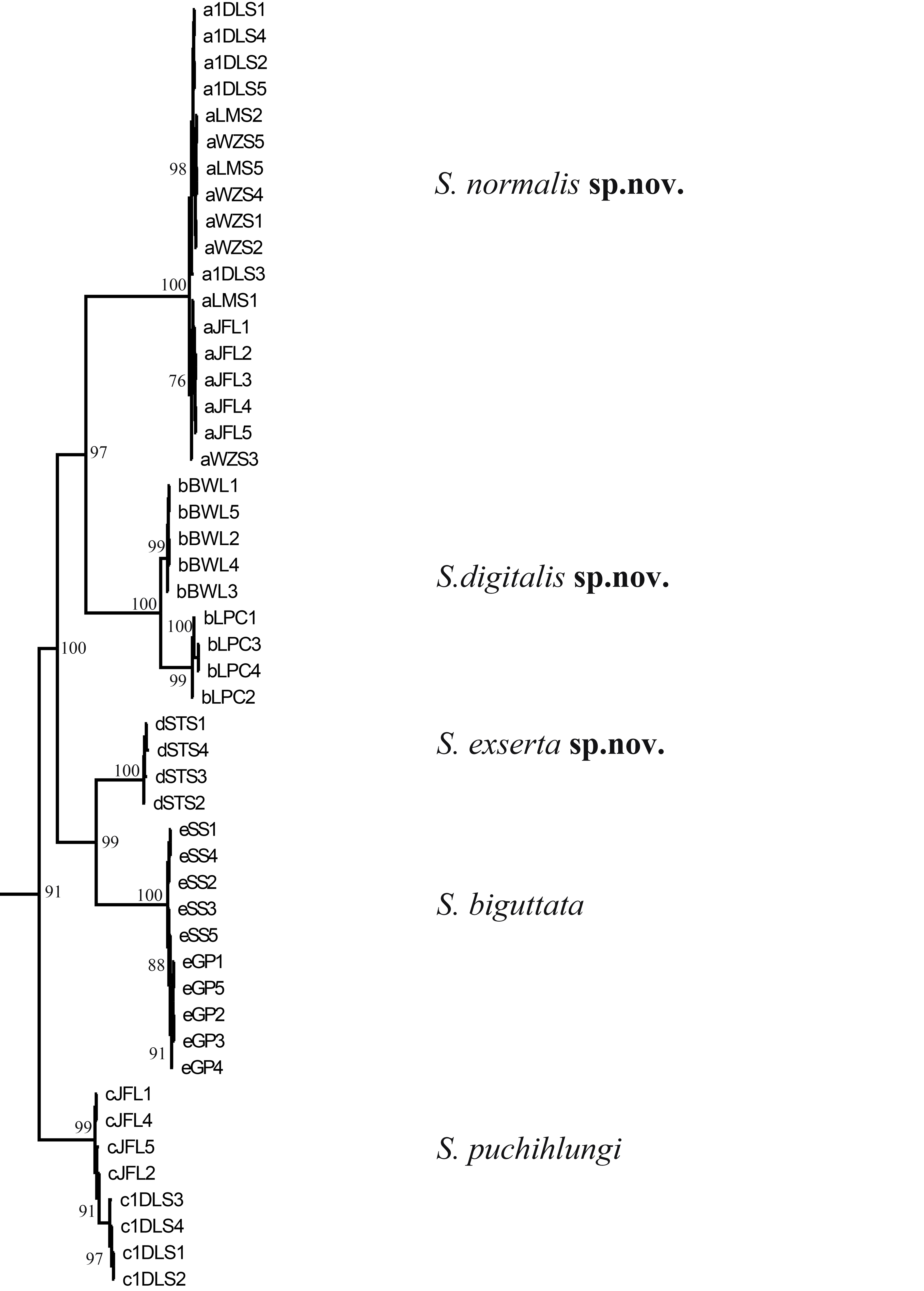

Supplement: S3 Fig — Outgroups are not shown. Numbers near node indicate the Bayesian posterior probabilities. (TIF) [file pone.0232821.s003.tif]

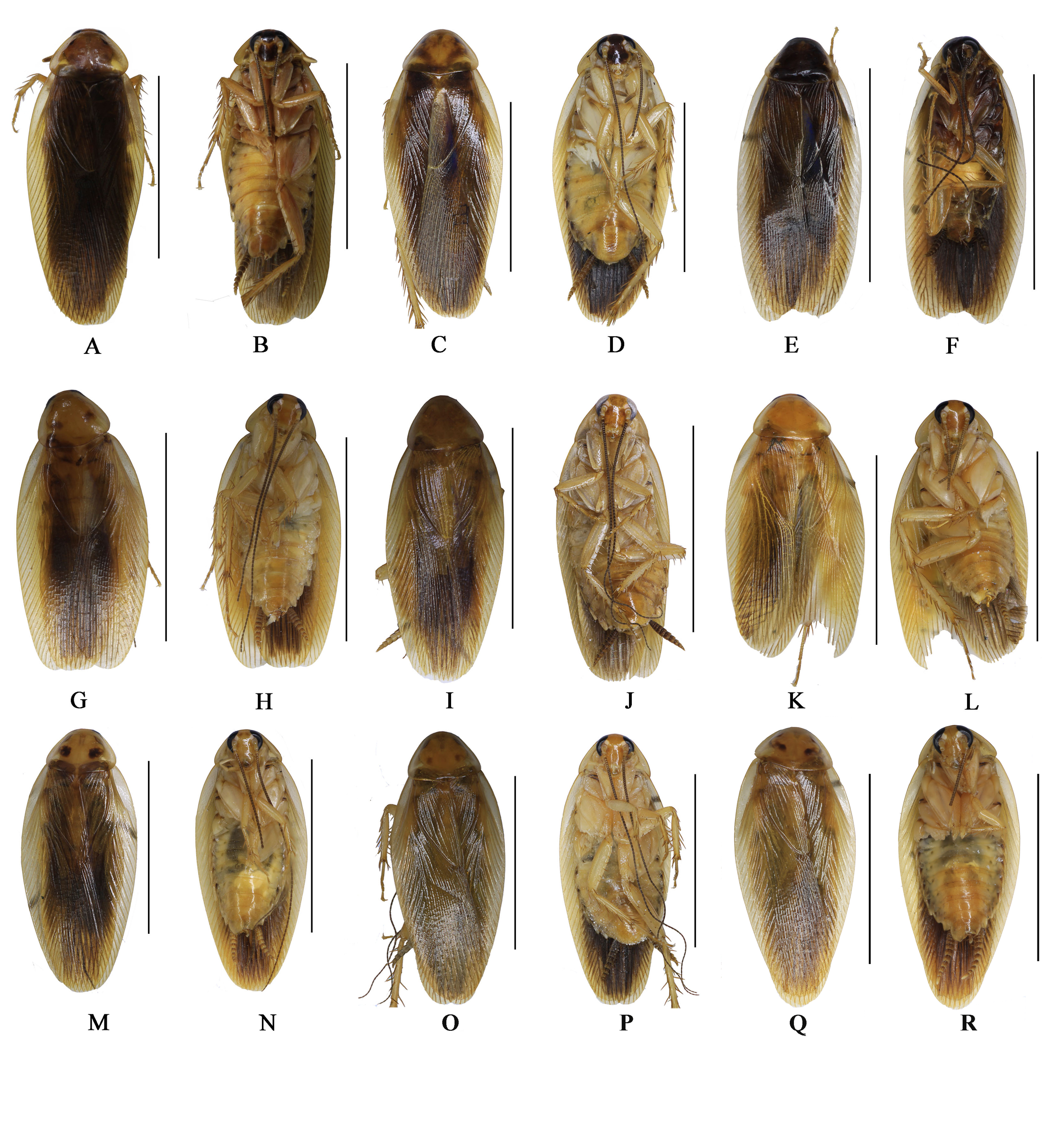

Supplement: S4 Fig — Sigmella puchihlungi: A–B (male, voucher cJFL5), C–D (female, voucher c3DLS2). S. normalis sp. nov.: E–F (male, voucher aWZS3). S. digitalis sp. nov.: G–H (male, voucher bBWL3), I–J (female, voucher bBWL2), K–L (male, voucher bLPC4). S. exserta sp. nov.: M–N (male, voucher dSTS4). S. schenklingi biguttata: O–P (female, voucher eSS1), Q–R (male, voucher eGP5). (A, C, E, G, I, K, M, O, Q) dorsal view; (B, D, F, H, J, L, N, P, R) ventral view; scale = 1cm. (TIF) [file pone.0232821.s004.tif]
