# Supplementary material for: Species diversity revealed in Sigmella Hebard, 1929 (Blattodea, ectobiidae) based on morphology and four molecular species delimitation methods
Source: PLoS One. 2020 Jun 10;15(6):e0232821. doi: 10.1371/journal.pone.0232821 (PMC7286484; doi:10.1371/journal.pone.0232821)
Supplement: S1 Table — (DOCX) [file pone.0232821.s005.docx]

**S1 Table.** K2P genetic distances among 5 *Sigmella* morphospecies.

| Morphospecies | K2P genetic distances | | | |
| --- | --- | --- | --- | --- |
| *S. puchilungi* |  |  |  |  |
| *S. normalis* sp.nov. | 0.1483 |  |  |  |
| *S. digitalis* sp.nov. | 0.1459 | 0.1499 |  |  |
| *S. exserta* sp.nov. | 0.1069 | 0.1352 | 0.1399 |  |
| *S. biguttata* | 0.1275 | 0.1659 | 0.1455 | 0.1360 |
