# Supplementary material for: Species diversity revealed in Sigmella Hebard, 1929 (Blattodea, ectobiidae) based on morphology and four molecular species delimitation methods
Source: PLoS One. 2020 Jun 10;15(6):e0232821. doi: 10.1371/journal.pone.0232821 (PMC7286484; doi:10.1371/journal.pone.0232821)
Supplement: S2 Table — (DOCX) [file pone.0232821.s006.docx]

**S2 Table.** K2P genetic distances within 5 *Sigmella* morphospecies.

| Morphospecies | K2P genetic distances |
| --- | --- |
| *S. puchilungi* | 0.0121 |
| *S. normalis* sp.nov. | 0.0055 |
| *S. digitalis* sp.nov. | 0.0241 |
| *S. exserta* sp.nov. | 0.0008 |
| *S. biguttata* | 0.0011 |
