# Supplementary material for: Species diversity revealed in Sigmella Hebard, 1929 (Blattodea, ectobiidae) based on morphology and four molecular species delimitation methods
Source: PLoS One. 2020 Jun 10;15(6):e0232821. doi: 10.1371/journal.pone.0232821 (PMC7286484; doi:10.1371/journal.pone.0232821)
Supplement: S3 Table — (DOCX) [file pone.0232821.s007.docx]

**S3 Table.** K2P genetic distances within 6 *Sigmella* MOTUs.

| MOTUs | K2Pgenetic distances |
| --- | --- |
| *S. puchilungi* | 0.0120 |
| *S. normalis* sp.nov. | 0.0055 |
| *S. digitalis* sp.nov. Ia1 | 0 |
| *S. digitalis* sp.nov. IIa1 | 0.0051 |
| *S. exserta* sp.nov. | 0.0008 |
| *S. biguttata* | 0.0011 |
